# Acute kidney injury is a common complication in children and adolescents hospitalized for diabetic ketoacidosis

**DOI:** 10.1371/journal.pone.0239160

**Published:** 2020-10-07

**Authors:** Shih-Kang Huang, Chi-Yu Huang, Chao-Hsu Lin, Bi-Wen Cheng, Ya-Ting Chiang, Yi-Chen Lee, Shu-Nin Yeh, Chon-In Chan, Wei-Kian Chua, Yann-Jinn Lee, Wei-Hsin Ting

**Affiliations:** 1 Department of Pediatric Endocrinology, MacKay Children’s Hospital, Taipei, Taiwan; 2 Department of Medicine, MacKay Medical College, New Taipei City, Taiwan; 3 Department of Pediatric Endocrinology, Hsinchu MacKay Memorial Hospital, Hsinchu City, Taiwan; 4 Department of Biological Science and Technology, National Chiao-Tung University, Hsinchu City, Taiwan; 5 Department of Pediatric Endocrinology, Ditmanson Medical Foundation Chia-Yi Christian Hospital, Chiayi, Taiwan; 6 Department of Pediatrics, Saint Paul’s Hospital, Taoyuan, Taiwan; 7 Department of Pediatrics, Centro Hospitalar Conde de São Januário, Macau, Taiwan; 8 Department of Medical Research, Tamsui MacKay Memorial Hospital, New Taipei City, Taiwan; 9 Department of Pediatrics, School of Medicine, College of Medicine, Taipei Medical University, Taipei, Taiwan; 10 Institute of Biomedical Sciences, MacKay Medical College, New Taipei City, Taiwan; 11 MacKay Junior College of Medicine, Nursing and Management, New Taipei City, Taiwan; International University of Health and Welfare, School of Medicine, JAPAN

## Abstract

Diabetic ketoacidosis (DKA) is associated with dehydration and which can cause acute kidney injury (AKI). The proportion of AKI in children and adolescents with DKA has not been reported in East Asian population. This study aimed to identify the prevalence of AKI and to determine whether there is an association between AKI severity and recovery time from metabolic acidosis in children and adolescents with DKA. Medical records of children and adolescents (aged <18 years) presenting with type 1 or type 2 diabetes mellitus and DKA between 2000–2017 at the MacKay Children’s Hospital were retrospectively reviewed. AKI was defined by an admission creatinine level >1.5 times the calculated expected baseline creatinine level. Patients were divided into three groups based on AKI severity: no AKI, mild AKI, and severe AKI. In total, 170 (56.5%) patients with DKA presented AKI (mild AKI, 116 [38.5%]; severe AKI, 54 [18.0%]). Heart rate and laboratory parameters related to dehydration, such as corrected sodium level and blood urea nitrogen, were strongly associated with AKI development (*P*<0.01). Blood pH, plasma glucose, and potassium levels were also associated with AKI. A negative correlation with borderline significance between the estimated glomerular filtration rate (eGFR) and recovery time from metabolic acidosis was observed in the severe AKI group. AKI was highly prevalent in children and adolescents with DKA. An association between AKI and biomarkers indicating dehydration was noted. The recovery time from metabolic acidosis following treatment may be longer in children with a decreased eGFR who present with severe AKI. AKI is a common complication in children with DKA.

## Introduction

Children with diabetic ketoacidosis (DKA) experience varying degrees of dehydration and electrolyte imbalance due to osmotic diuresis [1, 2]. Persistent polyuria is the most common symptom in children with DKA and can lead to progressive dehydration [3]. The compensatory mechanism for dehydration in children has not been well established; thus, children are more vulnerable to volume depletion than adults [4]. Prerenal acute kidney injury (AKI) may occur, and acute tubular necrosis may subsequently develop in patients with severe volume depletion [5–7]. When the glomerular filtration rate decreases, renal acid excretion becomes inadequate, and thus metabolic acidosis may occur [8]. As the normalization of metabolic acidosis is among the goals of DKA treatment [9], the presence of AKI may hinder a patient’s recovery from metabolic acidosis. Moreover, AKI is an independent factor associated with longer hospital stay and higher mortality rate in children [10].

Nevertheless, the prevalence and associated risk factors for AKI in children and adolescents with DKA in the East Asian population have not been reported, and the relationship between recovery time from metabolic acidosis and AKI severity in children with DKA has not been fully established. Therefore, our study aimed (1) to identify the prevalence of AKI and to analyze clinical and laboratory markers associated with AKI in children and adolescents hospitalized for DKA and (2) to determine whether an association between AKI severity and recovery time from metabolic acidosis exists during the treatment course of DKA.

## Methods

### Data collection

The medical charts of 558 DKA episodes recorded from 2000 to 2017 at the MacKay Children’s Hospital were retrospectively reviewed. Children aged <18 years with DKA as defined by the International Classification of Diseases (ICD-9 and ICD-10) coding for DKA (ICD-9 code 250.10 to 250.13 or ICD-10 codes E10.1 and E11.1) were included in the analysis. We obtained the following information from patients’ medical charts: age at diagnosis, sex, body weight, body height, heart rate, type of diabetes mellitus (DM), episodes of DKA, blood gas composition, and levels of blood glucose, corrected sodium, potassium, blood urea nitrogen, creatinine, and hematocrit at admission. The urine albumin to creatinine ratio (UACR) before admission or just after the hospitalization was also collected, to determine the presence of microalbuminuria [11]. The duration of hospitalization and the recovery time from metabolic acidosis (defined as the time from the beginning of treatment up to blood gas analysis showing pH >7.3 and bicarbonate level >15 mEq/L) were also recorded. Moreover, each patient’s estimated glomerular filtration rate (eGFR) at admission was calculated using the Schwartz formula [12] (eGFR = K × height in cm/creatinine in mg/dL; age <2 years, K = 0.45; age between 2 and 13 years, K = 0.55; boys aged ≥13 years, K = 0.7; girls aged ≥13 years, K = 0.55). Ethical approval was confirmed by the MacKay Memorial Hospital Institutional Review Board (MMH IRB No.: 14MMHIS267). Written informed consent was obtained from every patient or his/her guardian.

### Case definition and exclusion criteria

DKA was defined as hyperglycemia (blood glucose level ≥200 mg/dL) and low pH (pH ≤7.3) or bicarbonate (≤15 mEq/L) levels with elevated urine or serum ketones. Those who did not meet the criteria for DKA or did not have type 1 or type 2 DM were excluded. Furthermore, patients who were transferred from other hospitals where the initial management of DKA was performed, those who were coded with the wrong ICD code, those lacking important clinical information (e.g., serum creatinine level, body height), and those whose medical records were not available or missing were excluded.

The serum creatinine criteria of the Kidney Disease/Improving Global Outcomes (KDIGO) defined AKI as a creatinine level >1.5 times the baseline creatinine level [13]. Because there was no baseline creatinine data on the patients before admission, we calculated the expected baseline creatinine (EBC) levels using an eGFR [14, 15] of 120 mL/min/1.73 m^2^ and patients’ body height (Schwartz formula) [12]. We defined stage 0 AKI as a creatinine level <1.5 times the EBC, stage 1 AKI as creatinine level 1.5 to 2 times the EBC, stage 2 AKI as creatinine level 2 to 3 times the EBC, and stage 3 AKI as creatinine level >3 times the EBC. Patients were further divided into three groups: no AKI (stage 0 AKI), mild AKI (stage 1 AKI), and severe AKI (stage 2 and 3 AKI); stages 2 and 3 AKI were associated with higher mortality rates in children [16, 17]. Corrected sodium (Na) was calculated using the following formula: corrected Na = Serum Na + (1.6 mEq/L Na^+^ for every 100 mg/dL glucose in excess of 100) [18]. The microalbuminuria group was defined as UACR between 30–299 mg/g [11]. Recurrent DKA was defined as more than one episode of DKA.

### Patient treatment

Initially, patients received a bolus of 20 mL/kg isotonic saline at the emergency department or ordinary ward after a diagnosis of DKA was given. Thereafter, intravenous solution was administered at 1.5 times the maintenance rate for at least 24 hours; intravenous solution with different sodium and potassium concentrations was used, depending on the corrected serum sodium and potassium levels. Regular insulin was infused via the intravenous route at a rate of 0.1 U/kg/h. Blood glucose was monitored using the finger-stick blood sampling every hour after the start of treatment. Blood gas and electrolyte levels were generally checked every 6 hours or monitored according to each patient’s clinical condition. Once the blood glucose fell below 250 mg/dL, intravenous glucose concentration was gradually increased up to 10 g/dL. When blood glucose continued to fall below 120 mg/dL, we gradually decreased the insulin infusion rate and shifted to subcutaneous insulin injection after full correction of DKA.

### Statistical analysis

Continuous variables are expressed as medians and interquartile ranges (IQR). Categorical variables are expressed as numbers and percentages. Categorical variables were compared using the chi-square test or Fisher’s exact test in case of expected frequencies <5; continuous variables were compared among three or more groups using the Kruskal-Wallis test. After excluding the extreme value, a linear regression model was employed to explore the association between eGFR and recovery time from metabolic acidosis. All statistical analyses were performed using SAS, version 9.4 (SAS Institute, Inc., Cary, NC). A *P* value <0.05 was considered statistically significant.

## Results

In total, 558 DKA episodes were identified from February 1, 2000 to December 31, 2017. Of these DKA episodes, 257 were excluded as 115 did not meet the criteria for DKA, 36 came from other hospitals where the initial DKA management was performed, 46 had the wrong ICD code, 50 lacked important clinical data, and 10 had unavailable or missing medical records. Finally, 223 patients with 301 DKA episodes were included in this study.

Demographic characteristics, biological parameters, and AKI severity are summarized in Table 1.

**Table 1:** Clinical characteristics of children aged <18 years with diabetic ketoacidosis, classified by severity of acute kidney injury.

Clinical characteristics at admission	No AKI	Mild AKI	Severe AKI	Total	*P*-value
	N = 131	N = 116	N = 54	N = 301	
	(43.5%)	(38.5%)	(18.0%)		
Age, median (IQR), years	11.4 (8.7–14.3)	12.7 (8.0–16.0)	10.7 (7.3–13.7)	11.8 (8.5–15.4)	0.07
Sex, males, no. (%)	63 (48.1)	45 (38.8)	22 (40.7)	130 (43.2)	0.31
Type 1 DM, no. (%)	127 (96.9)	114 (98.3)	52 (96.3)	293 (97.3)	0.71
Type 2 DM, no. (%)	4 (3.1)	2 (1.7)	2 (3.7)	8 (2.7)	0.71
Newly diagnosed DM, no. (%)	78 (59.5)	50 (43.1)	26 (48.2)	154 (51.2)	0.03
Microalbuminuria, no. (%)	2 (1.5)	3 (2.6)	0 (0)	5 (1.7)	0.47
Recurrent DKA no. (%)	31 (23.7)	37 (31.9)	10 (18.5)	78 (25.9)	0.13
Initial physical examination, median (IQR)					
Body weight Z-score	-0.6 (-1.2–0)	-0.5 (-1.2–0.2)	-0.9 (-1.3–-0.1)	-0.5 (-1.2–0.1)	0.26
Body height Z-score	0.2 (-0.7–0.9)	0.05 (-0.9–0.5)	0 (-1.1–0.9)	0.1 (-0.8–0.7)	0.22
BMI Z score	-0.8 (-1.4–-0.1)	-0.4 (-1.1–0.2)	-1.0 (-1.6–-0.1)	-0.7 (-1.35–0)	0.03
Body surface area, m^2^	1.20 (1.0–1.4)	1.31 (0.9–1.5)	1.17 (0.8–1.4)	1.23 (0.9–1.5)	0.07
Heart rate, beats/min	106 (95–120)	122 (100–136)	135 (120–148)	118 (100–132)	<0.01
Initial laboratory values, median (IQR)					
pH	7.20 (7.10–7.26)	7.13 (7.04–7.22)	7.15 (7.03–7.22)	7.17 (7.06–7.23)	<0.01
Bicarbonate, mEq/L	9.0 (5.4–12.5)	7.0 (4.0–10.0)	7.1 (4.2–9.6)	7.6 (4.9–11.0)	<0.01
BUN, mg/dL	13.5 (11.0–18.0)	17.0 (13.0–20.0)	25.0 (19.0–36.0)	16 (12–21.5)	<0.01
Creatinine, mg/dL	0.8 (0.7–1.0)	1.1 (1.0–1.3)	1.5 (1.3–1.7)	1 (0.8–1.3)	<0.01
Na (corrected), mEq/L	139.1 (136.6–142.7)	140.8 (137.4–143.1)	142.4 (139.4–145.8)	140.6 (137.3–143.3)	<0.01
K, mEq/L	4.2 (3.8–4.8)	4.6 (4.1–5.2)	5.1 (4.8–5.8)	4.5 (4.0–5.2)	<0.01
Glucose, mg/dL	428 (354–528)	540 (431–627)	738 (576–861)	502 (409–638)	<0.01
Hct, %	44.0 (40.9–46.7)	45.5 (41.7–48.3)	43.9 (41.5–47.4)	44.3 (41.5–47.6)	0.17
HbA1c, %	13.4 (11.9–14.8)	12.5 (10.9–14.4)	12.4 (10.6–14.2)	12.8 (11.1–14.4)	<0.01
eGFR, mL/min/1.73 m^2^	94.4 (87.2–110.9)	70.5 (65.5–74.7)	53.0 (47.9–56.6)	76.1 (64.4–92.1)	<0.01

AKI: acute kidney injury; IQR: interquartile range; eGFR: estimated glomerular filtration rate

Hct: hematocrit; HbA1c, glycosylated hemoglobin; BUN, blood urea nitrogen

Patients had a median age of 11.8 (IQR, 8.5–15.4) years. Of the 301 DKA episodes, 130 (43.2%) were observed in male patients, 293 (97.3%) in patients with type 1 diabetes, and 154 (51.2%) episodes in patients with newly diagnosed diabetes.

Microalbuminuria was present in 5 (1.7%) patients before or after admission, which was not associated with the severity of AKI (*P* = 0.47). This finding indicated that although 147 (48.8%) patients had previously diagnosed DM, only few patients had microalbuminuria and the AKI severity at admission was not affected by the baseline kidney status. Seventy-eight (25.9%) patients experienced recurrent DKA episodes. Of the patients with recurrent DKA episodes, 31 (23.7%) did not have AKI, 37 (31.9%) had mild AKI, and 10 (18.5%) patients had severe AKI. The distribution of the AKI group and recurrent episodes DKA showed no significant differences (*P* = 0.13).

AKI was present at the time of admission in 170 (56.5%) patients who had DKA episodes; mild and severe AKI was found in 116 (38.5%) and 54 (18.0%) of those with episodes, respectively. Notably, among those with DKA who also presented AKI, 31.8% (54/170) developed severe AKI. The proportion of patients with AKI was less in those with newly diagnosed diabetes than in those with a previous diagnosis of diabetes (49.4% vs. 63.9%, *P* = 0.01).

Table 1 also shows the difference in clinical and laboratory data for patients with varying degrees of AKI severity. Children with AKI had lower pH values (no vs. mild vs. severe AKI, 7.20 vs. 7.13 vs. 7.15, *P*<0.01) and lower bicarbonate levels (no vs. mild vs. severe AKI, 9.0 vs. 7.0 vs. 7.1 mEq/L, *P*<0.01). Those with more severe AKI also had a statistically significantly higher heart rate (none vs. mild vs. severe AKI, 106 vs. 122 vs. 135 beats/min, *P*<0.01), corrected serum sodium levels (none vs. mild vs. severe AKI, 139.1 vs. 140.8 vs. 142.4 mEq/L, *P*<0.01), blood urea nitrogen (none vs. mild vs. severe AKI, 13.5 vs. 17.0 vs. 25.0 mg/dL, *P*<0.01), serum potassium levels (no vs. mild vs. severe AKI, 4.2 vs. 4.6 vs. 5.1 mEq/L, *P*<0.01), and blood glucose levels (no vs. mild vs. severe AKI, 428 vs. 540 vs. 738 mg/dL, *P*<0.01).

The median recovery time from metabolic acidosis of all patients admitted for DKA was 14.8 h (IQR, 9.1–21.6) (Table 2).

**Table 2:** Recovery time from metabolic acidosis, classified by severity of acute kidney injury.

Clinical characteristics at admission	No AKI N = 108	Mild AKI N = 101	Severe AKI N = 50	Total N = 259	*P*-value
Recovery time from metabolic acidosis (hours, IQR)	14.8 (8.4–21.7)	15.7 (10.2–21.8)	13.6 (8.7–18.5)	14.8 (9.1–21.6)	0.45
Proportion of patients with recovery time from metabolic acidosis <24 h (%)	86 (79.6)	81 (80.2)	42 (84.0)	209 (80.7)	0.84

AKI: acute kidney injury; IQR: interquartile range

**Fig 1:**
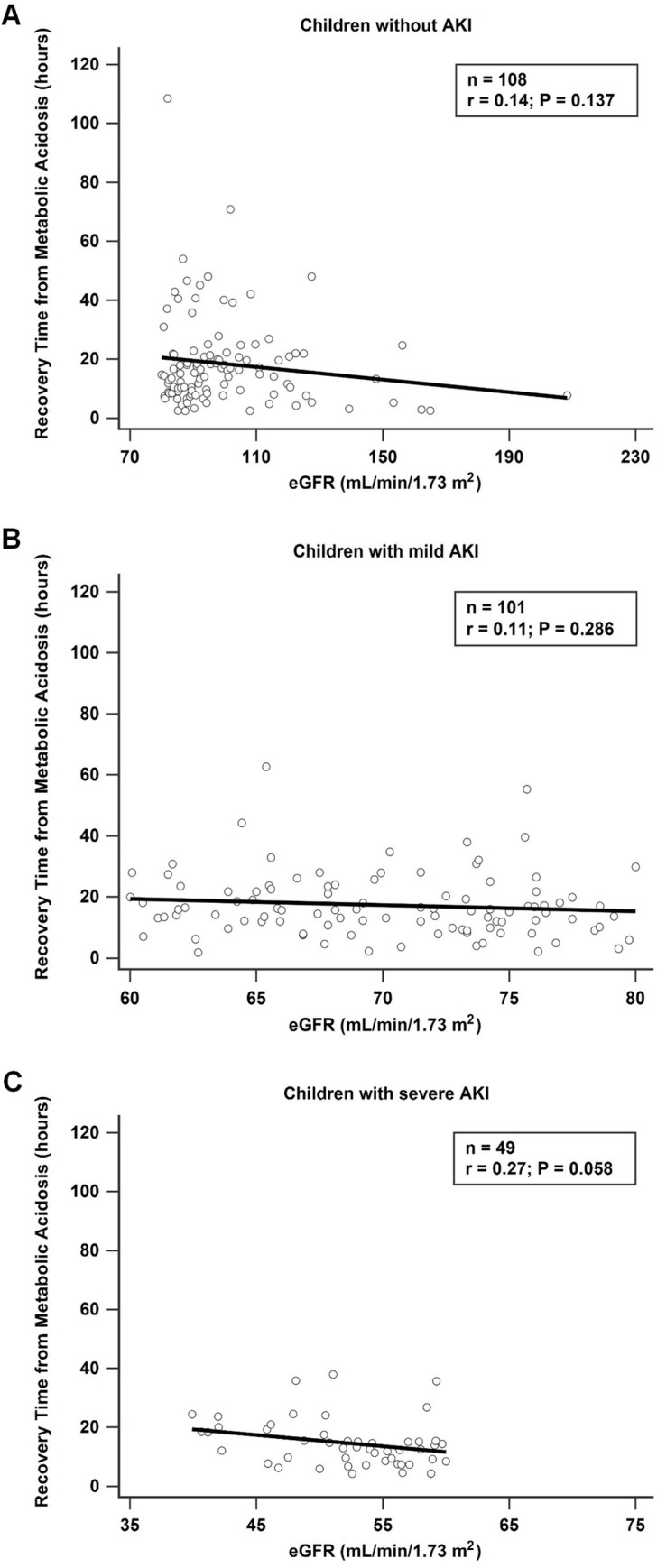
Linear regression showing differences between the estimated Glomerular Filtration Rate (eGFR) and recovery time from metabolic acidosis classified by Acute Kidney Injury (AKI) severity. (A) No association was observed between eGFR and recovery time from metabolic acidosis in children without AKI. (B) No association was observed between eGFR and recovery time from metabolic acidosis in children with mild AKI. (C) Borderline association was found between eGFR and recovery time from metabolic acidosis in children with severe AKI.

The recovery time from metabolic acidosis was 14.8 h (IQR, 8.4–21.7) in patients without AKI, 15.7 h (IQR, 10.2–21.8) in patients with mild AKI, and 13.6 h (IQR, 8.7–18.5) in patients with severe AKI; no statistically significant difference was observed across these groups. The proportion of patients with recovery time from metabolic acidosis <24 h was also not significantly different across the groups (none vs. mild vs. severe AKI, 79.6 vs. 80.2 vs. 84.0%, respectively, *P* = 0.84). However, in patients with severe AKI, a negative correlation (*R* = 0.27, *P* = 0.058) with borderline significance between eGFR and recovery time from metabolic acidosis was found (Fig 1C).

## Discussion

To our knowledge, this was the largest study to date that analyzed the prevalence and clinical markers of AKI in children and adolescents admitted for DKA, and it was the first study that focused on young East Asian patients. At the time of admission, 56.5% of children and adolescents with DKA developed AKI. Of note, one-third of children with AKI were classified as severe AKI, which indicated not only volume-responsive injury but also intrinsic renal tubular injury. Moreover, our study was the first study to show a borderline association between eGFR and recovery time from metabolic acidosis in patients with DKA presenting with severe AKI. Thus, only patients with DKA who had severe AKI may require a longer time to recover from metabolic decompensation status.

The reported prevalence of AKI in patients with DKA has been highly variable across different studies [19–22]. In the study of Orban et al., which included 94 patients, 50% of adults who were admitted for DKA met the RIFLE criteria for AKI [22]. Hursh et al. investigated 165 children admitted for DKA and reported a prevalence of AKI of 64.3% in children with DKA; here the AKI definition was based on the KDIGO criteria [19]. Both studies demonstrated that a high proportion of patients hospitalized for DKA developed AKI, which is consistent with our findings. In contrast, the prevalence of AKI in children with DKA was only 35.4% in India [20]; however, the baseline creatinine clearance they used to define AKI was not mentioned in their study. A recent study in Israel showed that the proportion of children with AKI who were admitted for DKA was only 30% [21], which is much lower than our result. One possible explanation for this discrepancy is that 80.5% of their study population had newly diagnosed type 1 DM, while in our study, only 51.2% of the patients had newly diagnosed diabetes. Furthermore, we found that the proportion of children with AKI was lower in children who were newly diagnosed with diabetes than in those who were previously diagnosed. Patients with repeated DKA episodes may have poor treatment compliance [23, 24], and their clinical condition, including the dehydration status, and biochemical abnormalities were possibly already worse when they started seeking medical intervention [25]. Additionally, different populations and criteria for diagnosing AKI in children may have also contributed to different results. It is worth noting that the serum creatinine level is an insensitive and delayed marker of impaired renal function [26]; thus, the kidneys may be injured before serum creatinine levels are elevated, which may imply that the actual prevalence of AKI in children with DKA might be higher than expected.

Our study revealed that a borderline correlation between eGFR and recovery time from metabolic acidosis existed in children with DKA who had severe AKI. The greater the decline in eGFR, the longer the time needed to recover from metabolic acidosis. Furthermore, our findings highlighted that renal tubular injury was present in children with DKA who had severe AKI and that their ability to compensate for metabolic acidosis also decreased as their renal function declined. A previous report [27] demonstrated that a lower pH levels at admission in adults was an independent predictor of the longer time needed for DKA resolution. Moreover, our finding emphasized that severe AKI development may contribute to longer recovery time from metabolic acidosis. As the normalization of metabolic acidosis is one of the goals of DKA treatment, clinicians should note that when children have stage 2 AKI or more, their recovery time from metabolic acidosis may be longer, and recovery depends on the decline in their eGFR. Thus, the presence of AKI should be assessed especially when children with DKA have prolonged metabolic acidosis [28, 29].

In our study, approximately 80% of children with DKA recovered from metabolic acidosis on the first day, regardless of AKI severity, after adequate hydration and insulin treatment, which is consistent with previous results [21]. Previous reports have shown that the recovery time from metabolic acidosis is associated with DKA severity and insulin dosage [30, 31]. Our study is the first to document that AKI severity in children with DKA was not statistically associated with the time to recovery from metabolic acidosis.

We also demonstrated that higher heart rate, higher levels of blood glucose and corrected sodium were associated with the severity of AKI. All these findings suggested that volume depletion plays an important role in AKI development in children with DKA. A previous study assumed that a prerenal mechanism is the cause of AKI in children with DKA [19]. A recent study showed that volume depletion is the causative etiology of AKI in children with DKA [21], which is consistent with our findings. We were unable to analyze the relationship between clinical assessment of dehydration and AKI severity because of the retrospective nature of our study, and the accuracy of the clinical assessment of dehydration may have been unreliable due to possible discrepancies between findings across different operators [32].

AKI has been identified as an independent factor that may increase morbidity and mortality in children [33]. Several studies have indicated there is a relationship between AKI and long-term renal injury [34–36]. Children with diabetes inherently have increased risk of diabetic nephropathy [37]; thus, monitoring AKI in this population is crucial. Nevertheless, whether a single AKI episode influences the development of further kidney disease in diabetic children remains unknown. Thus, further prospective studies focusing on the effect of AKI on the trajectory of renal disease in children with DM are warranted.

### Strengths and limitations

This study has some limitations. The major limitation of this study is its retrospective nature; the results depended on the accuracy and completeness of the medical records. Additionally, the creatinine level that we used to calculate eGFR via the Schwartz formula was measured using the Jaffe method. In children with metabolic ketoacidosis, the presence of acetoacetate may have resulted in falsely elevated serum creatinine levels if measured using the Jaffe method [38]. However, the Jaffe method is the most widely used approach to measure serum creatinine in our country and the only assay available that measures serum creatinine level at our institution. Moreover, the serum creatinine level is a delayed marker of kidney injury, and the proportion of true kidney injury may be higher than expected. Despite all these limitations, our study has several strengths. First, our study included a large sample size of pediatric patients who experienced DKA episodes and had the longest period of retrospective data collection compared with other studies. Moreover, the same DKA protocol was applied to all patients. Our results provided further evidence on the prevalence of AKI in children hospitalized for DKA.

## Conclusions

AKI is highly prevalent in children and adolescents admitted for DKA. The recovery time from metabolic acidosis may be longer with a decreased eGFR in children with stage 2 and stage 3 AKI. Most children (approximately 80%) recovered from metabolic acidosis on the first day of treatment. Clinical markers indicating volume depletion have a strong association with AKI severity. Clinicians should note that AKI is a common complication in children who are admitted for DKA.

## Abbreviations

AKI: acute kidney injury
DKA: diabetic ketoacidosis
DM: diabetes mellitus
EBC: expected baseline creatinine
eGFR: estimated glomerular filtration rate
IQR: interquartile range
